# From struggle to opportunity: Reimagining medical education in a pandemic era

**DOI:** 10.1007/s40037-022-00702-2

**Published:** 2022-03-14

**Authors:** Sarah Burm, Victoria Luong, Kori LaDonna, Bryce Bogie, Lindsay Cowley, Jennifer M. Klasen, Anna MacLeod

**Affiliations:** 1 Continuing Professional Development and Division of Medical Education, Dalhousie University, Halifax, Nova Scotia Canada; 2 Department of Innovation in Medical Education and Department of Medicine, University of Ottawa, Ottawa, Ontario Canada; 3 MD/PhD Program, Faculty of Medicine, University of Ottawa, Ottawa, Ontario Canada; 4 Ottawa Blood Disease Centre, Ottawa Hospital Research Institute, Ottawa, Ontario Canada; 5 Clarunis, Department of Visceral Surgery, University Center for Gastrointestinal and Liver Diseases, University Hospital Basel, Basel, Switzerland

**Keywords:** COVID-19, Critical pedagogy, Medical education

## Abstract

The COVID-19 pandemic has disrupted the international medical education community in unprecedented ways. The restrictions imposed to control the spread of the virus have upended our routines and forced us to reimagine our work structures, educational programming and delivery of patient care in ways that will likely continue to change how we live and work for the foreseeable future. Yet, despite these interruptions, the pandemic has additionally sparked a transformative impulse in some to actively engage in critical introspection around the future of their work, compelling us to consider what changes could (and perhaps should) occur after the pandemic is over. Drawing on key concepts associated with scholar Paulo Freire’s critical pedagogy, this paper serves as a call to action, illuminating the critical imaginings that have come out of this collective moment of struggle and instability, suggesting that we can perhaps create a more just, compassionate world even in the wake of extraordinary hardship.

## Introduction

Medical education faculty and staff have worked tirelessly to mitigate the impact of the COVID-19 pandemic on admission processes, clinical placements, examinations, and the sustainability of the medical workforce [1–3]. Undeniably, COVID-19 has demanded an ‘all hands-on deck’ response, and conversations occurring throughout the medical education community and across social media this past year suggest that those working and learning in medical education are shifting—and, in some cases, re-conceptualizing—their personal and professional roles and responsibilities [4].

To say our world is in a fragile state would be an understatement. Rising levels of inequalities, combined with rapid climate change and increased geopolitical tensions, have only exacerbated the pain and suffering being experienced on a global scale. Yet, something powerful seems to have emerged through this untethering experience: many are actively engaging in critical introspection, recognizing that the taken-for-granted ideas embedded in our learning environments and professional practice as ‘just the way it is’ are, in many instances, problematic, even harmful.

Paolo Freire, a ground-breaking scholar of education [5], is renowned for describing how humanity responds to moments of collective struggle, finding hope in seemingly hopeless situations, imagining a better future, and mobilizing change toward this better future. Drawing on key elements of his critical pedagogy, this paper serves as a call to action, inviting clinician educators, researchers, and administrators to consider what changes could (and perhaps should) occur within medical education once the pandemic is over.

We turn to Freire’s most celebrated text, *Pedagogy of the Oppressed* [5] to help us illuminate the possibilities for what our future could hold. Freire recognized the importance of autonomy, persons speaking for themselves, and the impact genuine dialogue can have on how individuals behave in the world as well as how they interact with others. Colleagues working in medical education have previously drawn on Freire’s work [6–12] to humanize the experience of care and reengage trainees and clinicians to the reason they entered medicine in the first place: to heal others. In a similar vein, we find ourselves asking: what does it mean to take a Freirean perspective to critically reimagine medical education in this pandemic era?

## Critical pedagogy: A paradigm of liberation and freedom

Certainly, COVID-19 is not the only catalyst for bringing clarity and urgency to the need for radical reimagination. Members of the medical education community were sounding alarms long before the pandemic about the present culture of medicine and healthcare delivery [13–16]. However, one of the most distinguishing features of Freire’s work is that it occurred under conditions of political and social strife, providing a useful lens through which we can examine this current historic moment.

At its simplest level, Freire’s pedagogy seeks to empower all to rise above “*the oppressor whose consciousness [we] have internalized*” [5, p. 48]. Let us begin by clarifying our use of the term ‘oppressor’ as we acknowledge that our choice in using such powerful language might invoke an uncomfortable, perhaps even defensive, response. For many, the term oppressor conjures up images of a person or group exerting authority or power over another. While this type of oppression can certainly (and regularly does) manifest throughout medical education [17, 18], expressions of oppression can additionally reveal themselves in seemingly unintentional and tacit ways: through our language choices; the physical, social, and increasingly virtual spaces we occupy and give value to; and the construction and perpetuation of ideological practices that position certain groups in society as subordinate to others. Experiences of oppression are deeply personal and may be amplified or reduced depending on an individual’s unique personal and professional experiences and the sociocultural categorizations (e.g., race, class, gender, sexual orientation, ability, etc.) with which one identifies [19, 20].

Another important hallmark of Freire’s critical pedagogy is the belief that social change requires questioning the status-quo in the name of achieving greater social justice, or as Freire might suggest, to *reclaim our humanity*. Proponents of Freire’s work have encouraged contemporary scholars to reimagine his ideas in response to present-day debates. These same calls reverberate across the medical community, urging clinicians to “*be advocates for their patients not just within the walls of the healthcare setting but also outside*”, and medical educators to “*engage in the development of curricula that foster reflection and dialogue about power differentials in healthcare systems*” [12, p. 241–42]. This remains challenging, however, as critical pedagogy has been critiqued for generating little traction outside the academy [21]. Freire’s early work in particular has faced scrutiny by feminist, Indigenous, and decolonial scholars for its “*fixation with class*” [22, p. 121] and failure to acknowledge how class intersects with, and reinforces, other forms of oppression such as sexism, racism, and colonialism [22–25].

We recognize the task that lies before us is both a complex and tremendous undertaking. Freire presents what is *possible*, not what is easy due to the ongoing impact of discriminatory laws and practices that continue to oppress certain social groups. We are proposing Freire as one possible lens that we can use to view the current moment and how we respond to it. We do not aim to imply that our perspective represents the diversity of the entire community; rather, we aim to demonstrate a means through which its individual members can reflect and act to create a better world.

Drawing on four key Freirean concepts: 1) Praxis; 2) Relevance; 3) Dialogue and 4) Radical transformation, we focus our imaginative capacities on some of the institutional structures and messages that are being illuminated and challenged during this period of collective human struggle. Specifically, we focus our attention on the realities of those pursuing education, research, and/or administrative pathways. This is not to say the examples we share will not resonate with those in predominantly clinical roles. However, we recognize that the high workload and emotional labour our clinical colleagues are presently confronting does not always afford the same level of malleability as those pursuing clinical service, research, and teaching roles. Nevertheless, it is our hope that exploring these issues through a Freirean lens may reveal deeper structural and policy changes needing to be re-envisioned or advocated for in medical education.

## Praxis

Recognizing the multiplicity of perspectives, identities, and roles each of us assume, Freire’s work encourages us to reflect upon the dominant discourses driving our professional culture, and to develop a critical consciousness of our individual and collective circumstances, asserting that “*authentic liberation—the process of humanization—is not another deposit to be made in men. Liberation is a praxis: the action and reflection of men and women upon their world in order to transform it*” [5, p. 79]. More precisely, praxis is a deliberate approach to action. As Kemmis and Smith (2008) elaborate,

*It is action that is ****morally committed****, and ****oriented and informed by traditions in a field****. It is the kind of action people are engaged in when they think about what their action will mean ****in the world****. Praxis is what people do when they take into account all the circumstances and exigencies that confront them at a particular moment and then, taking the broadest view they can of what it is ****best**** to do, they ****act*** [26, p. 4].

In an unintended way, the pandemic seems to have motivated us to engage in praxis, albeit in different ways and admittedly, with varying levels of consciousness and consequence. For many, the last year has heightened concern towards pre-existing systemic inequities like access to healthcare and health outcomes for different groups within society—motivating individuals to speak up about structural stigma and invest time and resources into building meaningful relationships within their communities to address identified needs [4, 27, 28]. For others, the pandemic has served as a catalyst to acknowledge and voice how deeply onerous learning and working in medicine and medical education can be [29, 30], and how easy it is to become complacent to these often harmful and invisible structures so steeped in our professional culture. We are too familiar with the epidemic of burnout and mental illness penetrating our organizations and institutions [31, 32]. Now, in the time of COVID-19, we must additionally acknowledge, and weigh, the multitude of loss being experienced by healthcare professionals, learners, and academic faculty members [33, 34]. At a philosophical level, we know the benefits of nurturing emotional and physical wellbeing and that failure is an inevitable part of learning [35]. Unfortunately, medicine’s professional cultures can make it difficult for these sentiments to translate into practice. Our training models and workloads seemingly perpetuate this notion that busyness is a badge of honour, that having our professional lives bleed into our private lives is par for the course [31]. But at what cost?

Perhaps, then, one of the hopeful elements surfacing because of the pandemic is permission to slow down, reimagine routines, enforce new boundaries, assess role priorities, and adopt work schedules that have the potential to protect, rather than hamper, personal and professional efficacy [4]. For some, this may mean carving out greater space for intellectual exploration, critical introspection, or advocacy work. For others, it might mean fiercely protecting the limited ‘free time’ they have to spend with loved ones or to engage in self-care. We encourage the medical education community to use this unprecedented moment in history to reflect upon how their future work can more equitably proceed, and how they can treat others, and themselves, more compassionately through that process.

## Relevance

Freire’s work on *relevance *urges us to actively confront how our current systems and structures contribute to, or prevent us from, seeing systemic barriers, with an eye to redistributing power in order to transform and dismantle unjust relations of exploitation. Freire believed that education can serve to liberate people, helping them to become critical, creative, free, active, and responsible members of society: “*To no longer be prey to [oppression], its force, one must emerge from it and turn upon it*” [5, p. 51]. Freire believed that people act on the issues that are of central importance to them, and that together, we can leverage strong feelings to bring about change. From a Freirean perspective, then, education work should start by identifying the issues that are of central concern to local people: issues that seemingly bring about excitement, hope, anxiety, even anger.

Certainly, COVID-19 has inspired strong feelings. One of the key challenges affecting us all has been the physical location of our work. Web-based videoconferencing has become, in a very short time, an essential tool in the design, delivery, and administration of medical education [1, 36, 37]. Limiting in-person learning and entry into clinical spaces has been one of the biggest adjustments. We acknowledge and applaud the tremendous efforts of those who have worked hard to ensure that technological solutions are in place to allow our interactions to be as close to ‘business as usual’ as possible. However, business as usual means attending to how physical and virtual spaces are designed and occupied, hosting meetings in our home offices, at our kitchen table, and even from quiet corners in bedrooms or basements. When our pre-pandemic professional and educational work took place in offices, classrooms, and clinical spaces, the setting for our daily interactions was familiar. Although the content of the interactions was fluid and evolving, the spaces within which they occurred—while certainly not neutral—were, for the most part, recognizable and professional.

More complicated, remote work (and videoconferencing from home more specifically) exposes issues of privilege, particularly with respect to class disparities. Our team has been reflecting on the ways in which home-based videoconferencing exposes domestic expressions of social class, including economic, social, and cultural capital [38]—in some instances, making disparities between participants clear. Expressions of habitus [39], embodied markers of social class, such as accent, posture, and ways of thinking and acting, were certainly present in our face-to-face work and learning pre-COVID-19. Videoconferencing technologies, however, expose us to a new set of data about the realities of our colleagues’ home lives that may have otherwise remained obscured.

## Dialogue

Remote work has additionally exposed the realities of balancing multiple roles. Many of us have been forced to navigate our working from home strategy amidst childcare, familial obligations, home schooling, and engagement with anti-racism work in response to the unjust tragedies spotlighted in the media. Stark gender disparities in career advancement and time for scholarly pursuits are surfacing in medicine and medical education, demonstrated by the escalating discourse about how the pandemic is disproportionally impacting women—especially women of colour who withstand the intersectional effects of racism and sexism [27, 40–42].

Although improving gender equity and inclusivity has previously been identified as a priority in medicine [43–45], the COVID-19 pandemic has amplified stress levels and has exposed the weight of invisible work that women shoulder. Entrenched in our societal values is the expectation for women to take on the responsibilities for childcare and domestic tasks—forcing many to prioritize caregiving responsibilities over professional advancement before, but especially during, the pandemic [46, 47]. Similarly, an assumption embedded in working from home is that all of us can freely, without distraction, join the Zoom meeting or take the impromptu video call. Yet, from what we have seen expressed across different social networking sites and within our respective professional spheres, this assumption is quite often false. To complicate matters further, many of us aim to separate our personal and professional lives, so examining and potentially exposing the raw edges of the unique circumstances each of us has endured during the pandemic to our preceptors, administrators, or colleagues (be it changing dynamics at home or the effects the pandemic has had on our psychological wellbeing) feels even more disruptive.

The medical education community now finds itself with new information to process, new questions to consider, and new emotional and logistical work related to structures of labour and reward. The challenge to build a just, egalitarian society—in our case, a more equitable and inclusive professional culture—is complex and beyond the scope of any individual. However, while no one has all the answers, no one is completely without ideas or suggestions, either. A critical feature of our work, then, must be to create the conditions for dialogue. Through dialogue, Freire writes,

*people develop their power to perceive critically the way they exist in the world with which and in which they find themselves; they come to see the world not as a static reality, but as a reality in process, in transformation *[5, p. 83]*.*

To discover genuine ways forward, all of us must act as both learners and teachers. Education must be a mutual learning process whereby we openly acknowledge and name the multiplicity of experiences individuals have endured, many of which remain invisible and frequently overlooked. Validating and normalizing, rather than avoiding and denying these experiences is one place for us to begin. Exposing the power dynamics that provoke different “*struggles among people oppressed differently*” [24, p. 453] and that render certain experiences invisible—a point which Freire, himself, was criticized for overlooking [24]—is another. Our job in medical education is to create the conditions in which genuine dialogue can occur without restraint; where we can listen to, and learn from, the experiences of others.

## Radical transformation

With this destabilizing force comes an opportunity to pause and carefully re-examine the entrenched social, institutional, and educational issues the pandemic has magnified. The global impact of the COVID-19 pandemic has meant that struggle, while certainly an individual experience, is also collective. COVID-19 has spotlighted longstanding cultural norms and social practices, forcing us to consider them through a more holistic and critical lens. While contemplating the fuzzy boundaries between home and work may have always been part of an individual’s considerations, COVID-19 has meant that institutions are also required to reconsider what is valued and why.

During challenging situations, we often become so consumed with mitigating struggle and alleviating discomfort that we overlook, using Freire’s words, opportunities for liberation [5]. Perhaps what emerges from the disruption of the pandemic is a radical reimagining of what counts within our respective spheres of influence. Of course, there is risk involved in overthrowing, or at the very least circumventing, role expectations and institutional structures rooted in tradition, especially for those who have experienced, and continue to confront, intersectional oppression [48]. Do we feel capable of taking such risks to create a more equitable and empathic system? Freire writes that “*to exist humanly is to name the world, to change it*” [5, p. 88]. We would add that the pursuit of full humanity additionally demands asking tough questions about the purposes of existing education and healthcare practices and policies. Ultimately, the Freirean goal of radical transformation aims to actively involve whole communities in transforming each individual’s life, but also the community and society as a whole. It is not an individualistic academic exercise, but a dynamic process in which education and development are intricately interwoven. By engaging in regular critical reflection [49], we become capable of effectively transforming daily life.

## Implications for policy and practice

At the time of writing this paper, more than 50% of the global population have received at least one dose of a COVID-19 vaccine. This provides some comfort that the pandemic’s end is in sight, but managing uncertainty is an inevitable part of being human. How will the medical education community move forward in an era of unprecedented complexity? More importantly, how can we move forward in a way that unabashedly shows our humanity?

For many, the pandemic era has shown that time is flexible, presence is malleable, and productivity ebbs and flows [4]. Perhaps, then, we need to re-envision what working amidst a global pandemic has come to mean and, more specifically, what we can realistically expect from people in a time of precarity. There are small changes that can be made immediately within medicine’s professional culture to cultivate a more autonomous learning and working environment. Where possible, encourage individuals to restructure their workdays around when they are most physically and cognitively productive. Re-consider whether time-specific attendance at meetings is required. Relatedly, departments and supervisors are urged to take advantage of different technological platforms not only to discuss professional tasks and obligations, but to additionally address individuals’ concerns around their professional future (e.g., graduation, promotion, job security) and to facilitate human connection around pandemic-induced occupational stress, such as extended periods of social isolation, changes to routine, interruptions to training, and delays in scholarly pursuits. Similarly, recognize that merely having access to physical space in which to work is an amenity that may not be consistently available to all, particularly for those who have not previously had the ability, or perhaps more accurately, the freedom, to work from home.

Other changes will take further deliberation and meaningful engagement with divergent audiences but are no less important. Modernize university tenure and promotions criteria to consider the variety of ways research, teaching, and service can be both “*intellectually rigorous and accessible to multiple audiences*” [21, p. 10]. Relatedly, we urge clinician educators and administrators to move beyond conversations around quotas to “fix” growing diversity concerns to naming and changing the forces that prevent underrepresented groups from feeling safe and supported.

As mentioned previously, clinical responsibilities are not flexible like academic work often is, and these proposed changes may not be realistic for everyone. Since the nature of day-to-day activities, suitability to distance work, and autonomy over workload differ significantly between medical specialties, we urge department chairs and division chiefs to re-consider their work practices with the perspectives and experiences of their colleagues in mind. This sentiment also applies to medical trainees who may be adjusting to evolving safety protocols and routines within the clinical setting while simultaneously experiencing emotional distress about lost or modified clinical experience and the impact of this on their futures [33, 34]. It is critical that those in leadership positions make time to understand their team’s sources of concern and meaningfully address these through institutional-wide measures that promote a culture of wellness, rather than intensify specific sources of anxiety and fear [50].

## Conclusion

Inspired by Freire to imagine a utopian future for the people of medical education, we have explored some of the institutional structures and messages that are being called into question during this period of collective human struggle. The pandemic has provided space for us to reimagine and advocate for a more humane institutional culture and environment, based not on depersonalization and surveillance, but rather one rooted in radical empathy and autonomy. The collective struggle of the pandemic makes space for us to re-examine our priorities from a critical perspective and, in embracing a Freirean approach, actively work towards dismantling oppressive practices. We recognize this re-calibration may feel somewhat uncomfortable. However, we hope it will eventually settle into a more sustainable rhythm of work, one where, in the post-pandemic future, we enthusiastically work to live rather than begrudgingly live to work.
